# Supplementary material for: The interactome of intact mitochondria by cross-linking mass spectrometry provides evidence for coexisting respiratory supercomplexes
Source: Mol Cell Proteomics. 2017 Dec 8;17(2):216–32. doi: 10.1074/mcp.RA117.000470 (PMC5795388; doi:10.1074/mcp.RA117.000470)
Supplement: Supplemental Data [file supp_RA117.000470_133922_0_supp_23978_fzffwf.zip › spectra_annotation/mito_DR_spectra_annotation/197-1-19-1-12-1.pdf]

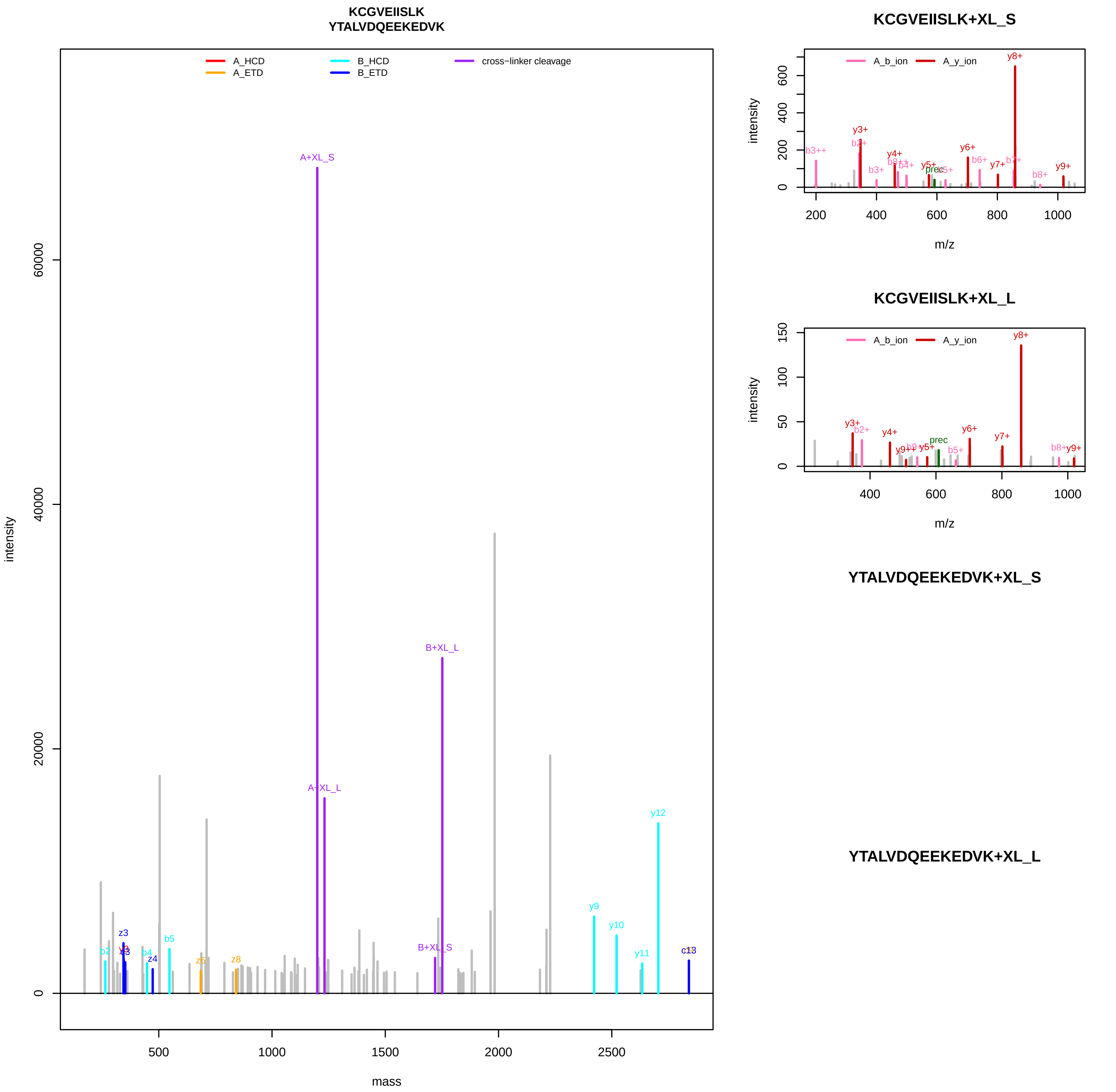

KCGVEIISLK
YTALVDQEEKEDVK
A_HCD
A_ETD
B_HCD
B_ETD
cross−linker cleavage
A+XL_S
A+XL_L
B+XL_L
B+XL_S
b2
y3
z3
c3
b4
z4
b5
z6
z8
y9
y10
y11
y12
c13
intensity
mass
0
20000
40000
60000
500
1000
1500
2000
2500
KCGVEIISLK+XL_S
A_b_ion
A_y_ion
b3++
y3+
b2+
b3+
y4+
b8++
b4+
y5+
prec
b5+
y6+
b6+
y7+
b7+
y8+
b8+
y9+
intensity
m/z
0
200
400
600
800
1000
KCGVEIISLK+XL_L
A_b_ion
A_y_ion
y3+
b2+
y4+
y9++
b9++
y5+
prec
b5+
y6+
y7+
y8+
b8+
y9+
intensity
m/z
0
50
100
150
400
600
800
1000
YTALVDQEEKEDVK+XL_S
YTALVDQEEKEDVK+XL_L
